# An Ir(III) Complex Photosensitizer With Strong Visible Light Absorption for Photocatalytic CO_2_ Reduction

**DOI:** 10.3389/fchem.2019.00259

**Published:** 2019-05-01

**Authors:** Yusuke Kuramochi, Osamu Ishitani

**Affiliations:** Department of Chemistry, Graduate School of Science and Engineering, Tokyo Institute of Technology, Tokyo, Japan

**Keywords:** strong visible-light absorption, metal complex, photocatalyst, electron donor, rhenium, CO_2_ reduction, photosensitizer, iridium

## Abstract

A cyclometalated iridium(III) complex having 2-(pyren-1-yl)-4-methylquinoline ligands [**Ir(pyr)**] has a strong absorption band in the visible region (ε_444nm_ = 67,000 M^−1^ cm^−1^) but does not act as a photosensitizer for photochemical reduction reactions in the presence of triethylamine as an electron donor. Here, 1,3-dimethyl-2-(o-hydroxyphenyl)-2,3-dihydro-1H-benzo[d]imidazole (BI(OH)H) was used instead of the amine, demonstrating that BI(OH)H efficiently quenched the excited state of **Ir(pyr)** and can undergo the photochemical carbon dioxide (CO_2_) reduction catalyzed by *trans*(Cl)-Ru(dmb)(CO)_2_Cl_2_ (dmb = 4,4′-dimethyl-2,2′-bipyridine, **Ru**) to produce formate as the main product. We also synthesized a binuclear complex combining **Ir(pyr)** and **Ru** *via* an ethylene bridge and investigated its photochemical CO_2_ reduction activity in the presence of BI(OH)H.

## Introduction

**Figure 1:**
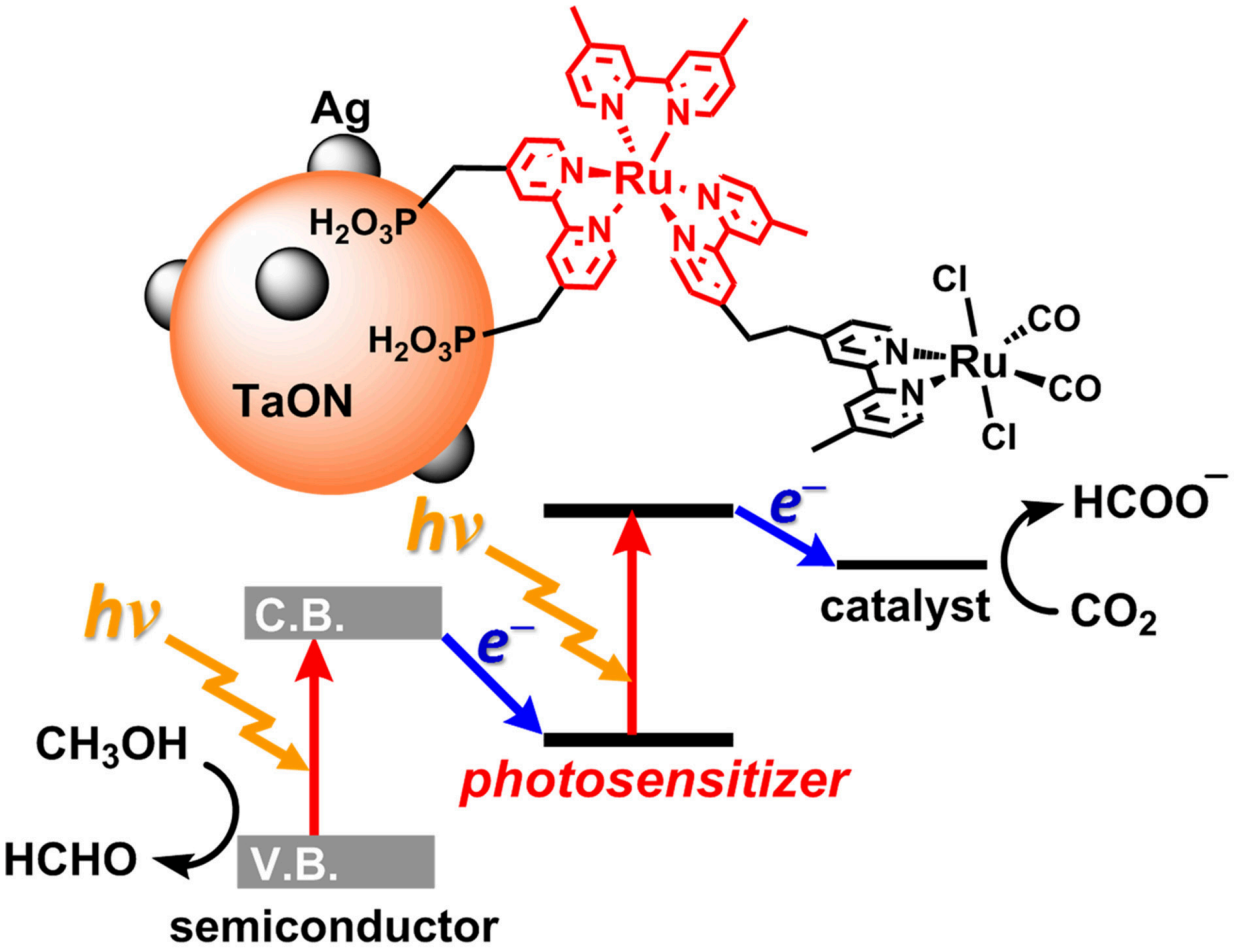
The artificial Z-scheme system for CO_2_ (carbon dioxide) reduction, consisting of a semiconductor (TaON) and a Ru(II) binuclear complex **(RuRu')** (Sekizawa et al., 2013).

Today, the consumption of fossil resources releases a tremendous amount of carbon dioxide (CO_2_), which has had a serious impact on global climate change. The reduction in fossil resources in the future will induce shortages in both energy and carbon sources. To resolve these serious problems, the development of alternative energy systems that produce reduced volumes of CO_2_ by using solar light as an energy source is desirable. To utilize a wider range of visible light from the sun, nature-inspired artificial Z-scheme systems have been developed by using semiconductors modified with metal complexes (Sato et al., 2011; Sekizawa et al., 2013; Kuriki et al., 2016, 2017; Sahara et al., 2016; Kumagai et al., 2017). Some metal complex photocatalytic systems that consist of a photosensitizer (PS) and a catalyst (CAT) can selectively induce CO_2_ reduction and suppress hydrogen (H_2_) evolution. These systems require a sacrificial electron donor due to the relatively low oxidation power of the PS unit in the excited state (Yamazaki et al., 2015; Tamaki and Ishitani, 2017; Kuramochi et al., 2018a) Step-by-step excitation of both the semiconductor and the metal complex produces an electron with high reducing power and a hole with high oxidizing power, allowing for CO_2_ reduction by weaker electron donors such as methanol (Figure 1; Sekizawa et al., 2013).

Ru(II) tris-diimine complexes [Ru(N^∧^N)_3_]^2+^ have been frequently used as the PS unit of supramolecular photocatalysts, which have strong absorption in the visible region, a long lifetime of the ^3^MLCT excited state, and a stable one-electron reduced state. However, [Ru(N^∧^N)_3_]^2+^ has a problem in that one of the N^∧^N ligands is relatively easily released during the photocatalytic reaction to give [Ru(N^∧^N)_2_(Solvent)_2_]^2+^-type complexes that work as catalysts for CO_2_ reduction (Lehn and Ziessel, 1990; Yamazaki et al., 2015; Kuramochi et al., 2018a). In addition, a PS unit with stronger absorption in the visible region compared to [Ru(N^∧^N)_3_]^2+^ should be more favorable for constructing new photocatalytic systems.

**Figure 2:**
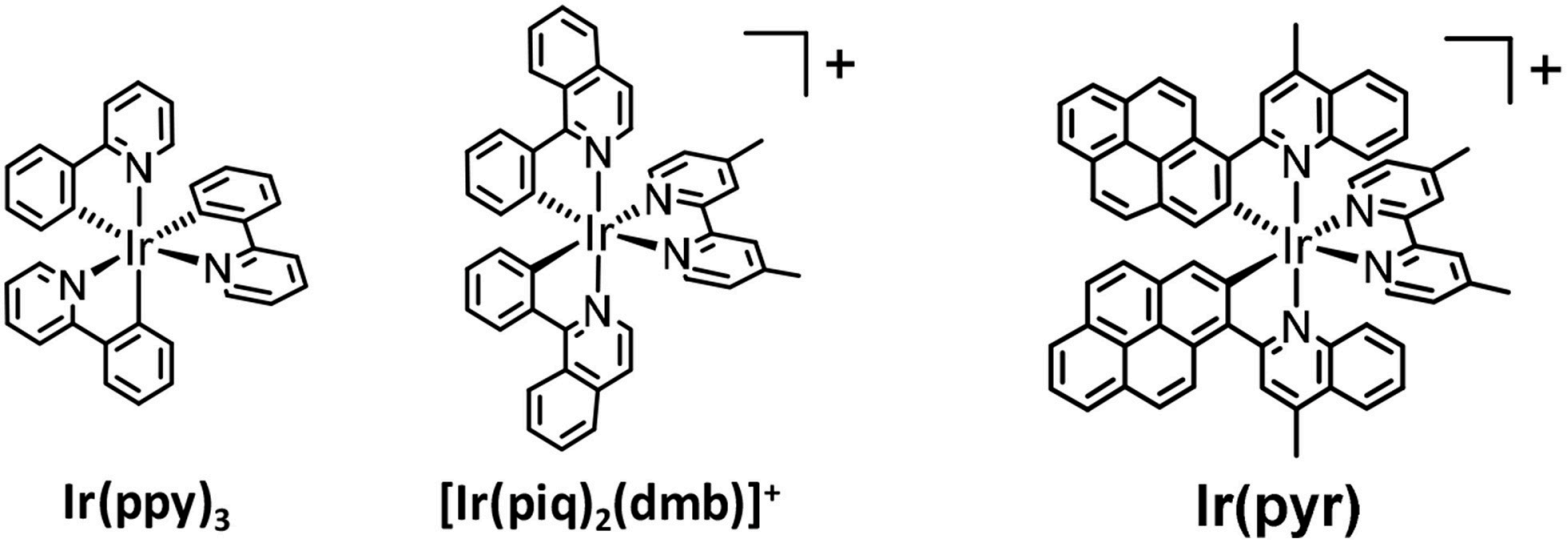
Structures of iridium(III) complexes.

In recent years, cyclometalated iridium(III) complexes, such as Ir(ppy)_3_ (ppy = 2-phenylpyridine) and [Ir(ppy)_2_(N^∧^N)]^+^, have been used as PSs in various photocatalytic reactions, such as H_2_ evolution (Goldsmith et al., 2005; Lowry and Bernhard, 2006), CO_2_ reduction (Thoi et al., 2013; Bonin et al., 2014; Chen et al., 2015; Rao et al., 2017, 2018), and organic synthesis (Figure 2; Prier et al., 2013; Schultz and Yoon, 2014; Shaw et al., 2016), even though the absorption by Ir(ppy)_3_ and [Ir(ppy)_2_(N^∧^N)]^+^ is relatively weak in the visible region. We already reported that [Ir(piq)_2_(dmb)]^+^ (piq = 1-phenylisoquinoline, dmb = 4,4′-dimethyl-2,2′-bipyridine), which has a stronger absorption in the visible region (ε_444nm_ = 7,800 M^−1^ cm^−1^) than Ir(ppy)_3_, acts as a PS for CO_2_ reduction without forming decomposed species that catalyze CO_2_ reduction (Figure 2; Kuramochi and Ishitani, 2016). The intense absorption at the longer wavelength allowed for the selective excitation of the PS without exciting the CAT, such as *fac*-Re(dmb)(CO)_3_Br (dmb = 4,4′-dimethyl-2,2′-bipyridine). In addition, the supramolecular photocatalyst, where [Ir(piq)_2_(BL)]^+^ (BL = bridging ligand) is connected with *fac*-Re(BL)(CO)_3_Br, works as a better photocatalyst for CO_2_ reduction compared to the mixed system of the corresponding mononuclear complexes, i.e., [Ir(piq)_2_(dmb)]^+^ and *fac*-Re(dmb)(CO)_3_Br. Although [Ir(piq)_2_(dmb)]^+^ has a stronger absorption in the visible region compared to Ir(ppy)_3_ and the advantages over [Ru(N^∧^N)_3_]^2+^ as mentioned above, its absorption in the visible region is weaker than that of [Ru(N^∧^N)_3_]^2+^. It has been reported that several Ir(III) complexes that have stronger absorption bands in the visible region than [Ru(N^∧^N)_3_]^2+^ can act as PSs for H_2_ evolution (Takizawa et al., 2012, 2014, 2018; Fan et al., 2014). Fan et al. reported that an Ir(III) complex with 2-(pyren-1-yl)-4-methylquinoline ligands [**Ir(pyr)**, Figure 2] showed a very strong absorption band in the visible region, ε(450 nm) > 60,000 M^−1^ cm^−1^. Unfortunately, it could not photocatalyze H_2_ evolution using K_2_PtCl_4_ as CAT in the presence of triethylamine (TEA). This inactivity was explained by the lack of photoinduced electron transfer from TEA to the excited state of **Ir(pyr)**, as the reduction potential of the excited state of **Ir(pyr)** is less than the oxidation potential of TEA (Fan et al., 2014). We reported that the Ru(II)–Ru(II) supramolecular photocatalyst can selectively reduce CO_2_ to formic acid (HCOOH) by using 1,3-dimethyl-2-(*o*-hydroxyphenyl)-2,3-dihydro-1H-benzo[d]imidazole (BI(OH)H) as an electron donor (ED) with a high turnover number (TON_HCOOH_) and a high quantum yield (Φ_HCOOH_; Tamaki et al., 2015). This photocatalytic reaction does not proceed in the absence of BI(OH)H even if triethanolamine (TEOA), which has a similar oxidation potential to TEA, is used. This is because BI(OH)H has a much stronger reducing power (*E*${}_{1/2}^{∙\text{x}}$ = +0.02 V *vs*. Ag/AgNO_3_) (Hasegawa et al., 2006; Elgrishi et al., 2017; Kuramochi et al., 2018a) than TEA (*E*${}_{\text{p}}^{∙\text{x}}$ = +0.67 V *vs*. Ag/AgNO_3_) (Yamazaki et al., 2015; Elgrishi et al., 2017).

**Scheme 1:**
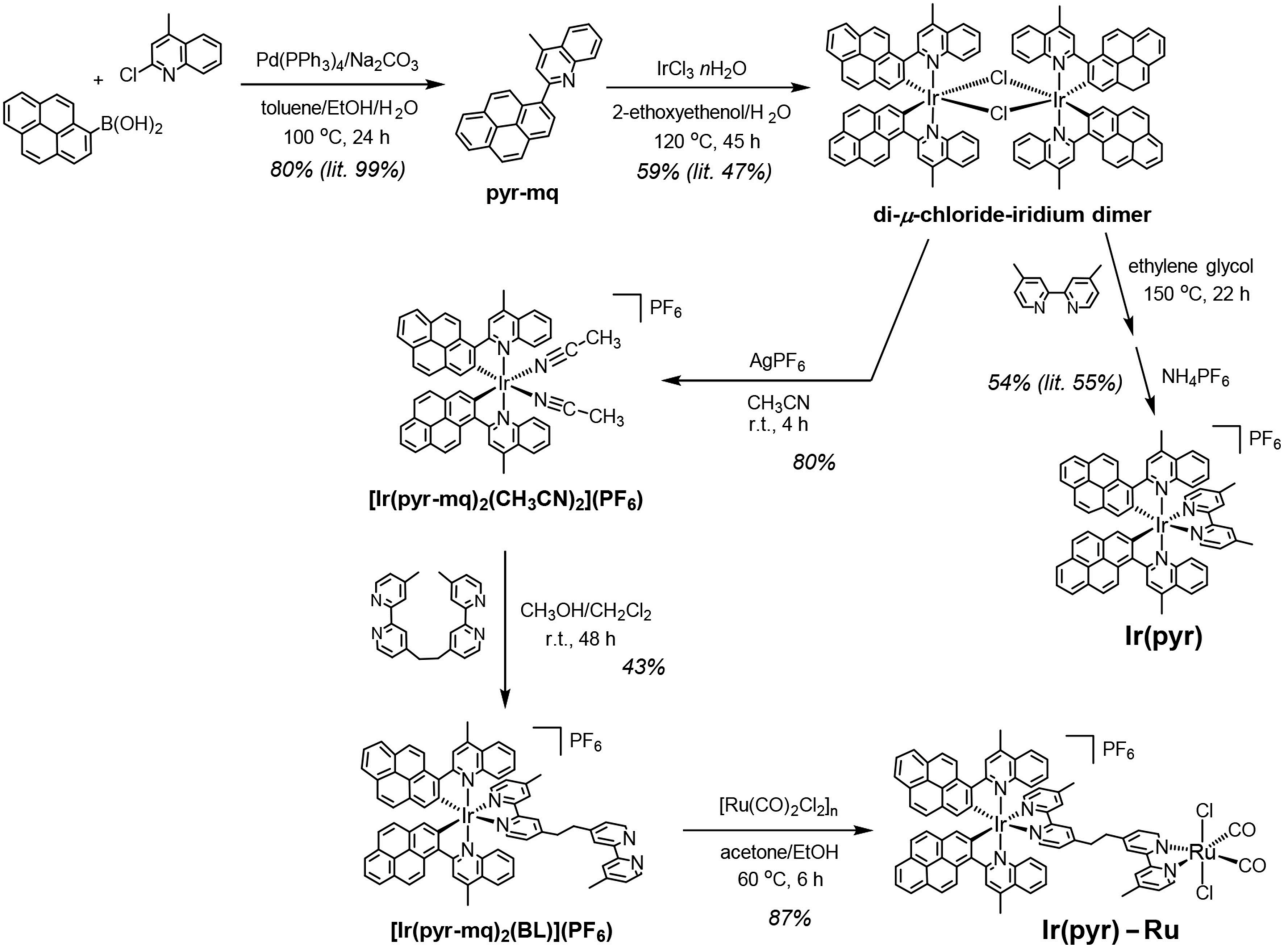
Synthetic routes to Ir(pyr)–Ru and Ir(pyr).

Herein, we report the successful use of **Ir(pyr)** as a PS for CO_2_ reduction by using BI(OH)H as ED and *trans*(Cl)-Ru(dmb)(CO)_2_Cl_2_ (**Ru**) as CAT. We also synthesized a supramolecular photocatalyst from **Ir(pyr)** (**Ir(pyr)–Ru**; Scheme 1) and investigated its photocatalytic activity for CO_2_ reduction.

## Results and Discussion

### Synthesis of the Binuclear Complex, Ir(pyr)–Ru

**Ir(pyr)–Ru** was synthesized according to Scheme 1. The di-μ-chloride-iridium dimer (Fan et al., 2014) was reacted with AgPF_6_ in acetonitrile to give the mononuclear acetonitrile–iridium complex. This complex was reacted with 1,2-bis(4′-methyl-[2,2′-bipyridin]-4-yl)ethane (BL), and the crude product was isolated using a silica gel column giving [Ir(pyr-mq)_2_(BL)](PF_6_) in 43% yield, based on the acetonitrile–iridium complex. This was reacted with [Ru(CO)_2_Cl_2_]_n_, which was pretreated by refluxing in the solvent (Kuramochi et al., 2015), giving the desired binuclear complex **Ir(pyr)–Ru** as a PF_6_ salt in 87% yield.

### Photophysical and Electrochemical Properties

**Figure 3:**
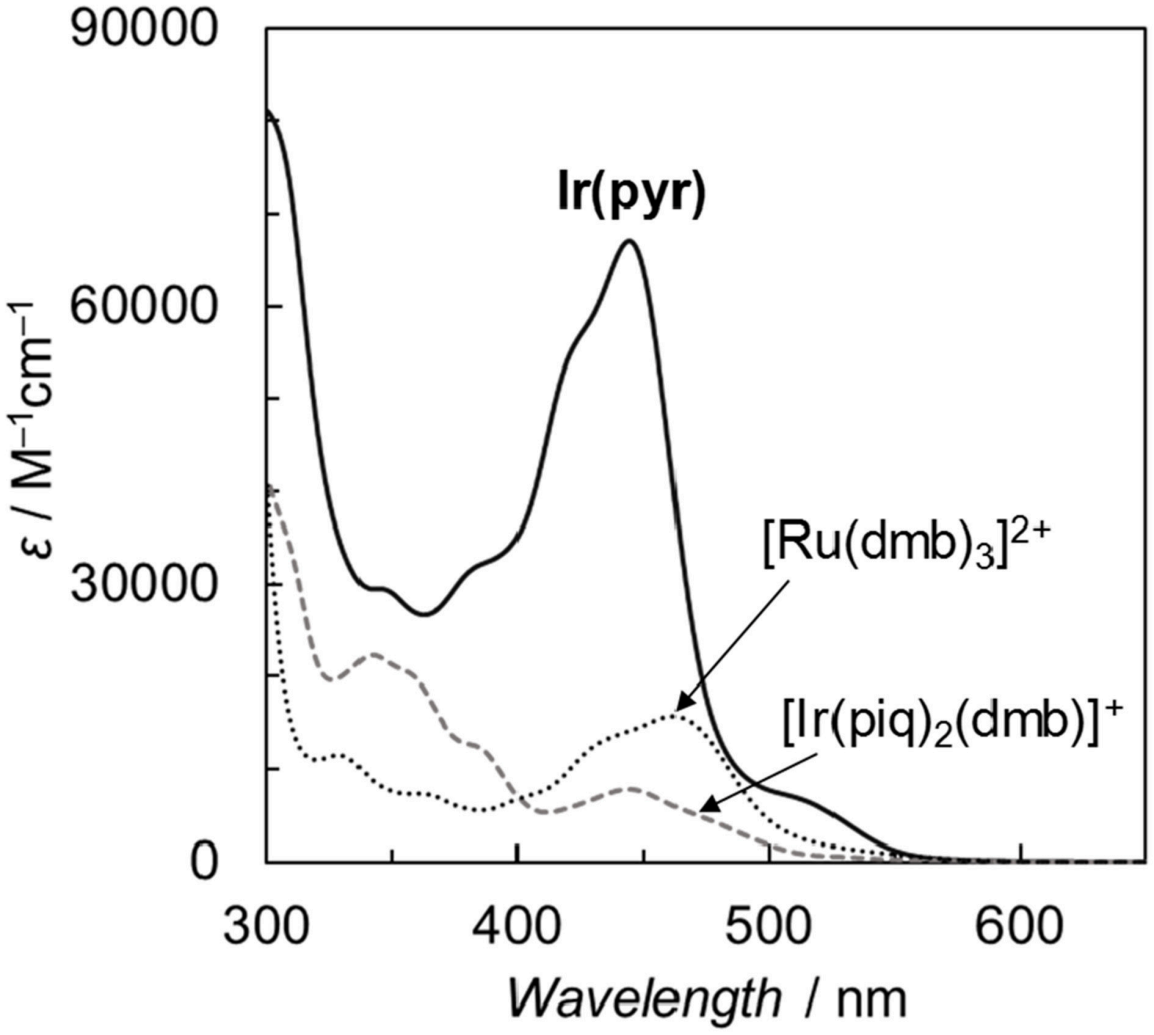
Comparison of the ultraviolet–visible (UV–vis) absorption spectra of **Ir(pyr)**, [Ir(piq)_2_(dmb)](PF_6_), and [Ru(dmb)_3_](PF_6_)_2_ in *N,N*-dimethylacetamide (DMA) at 298 K.

**Figure 4:**
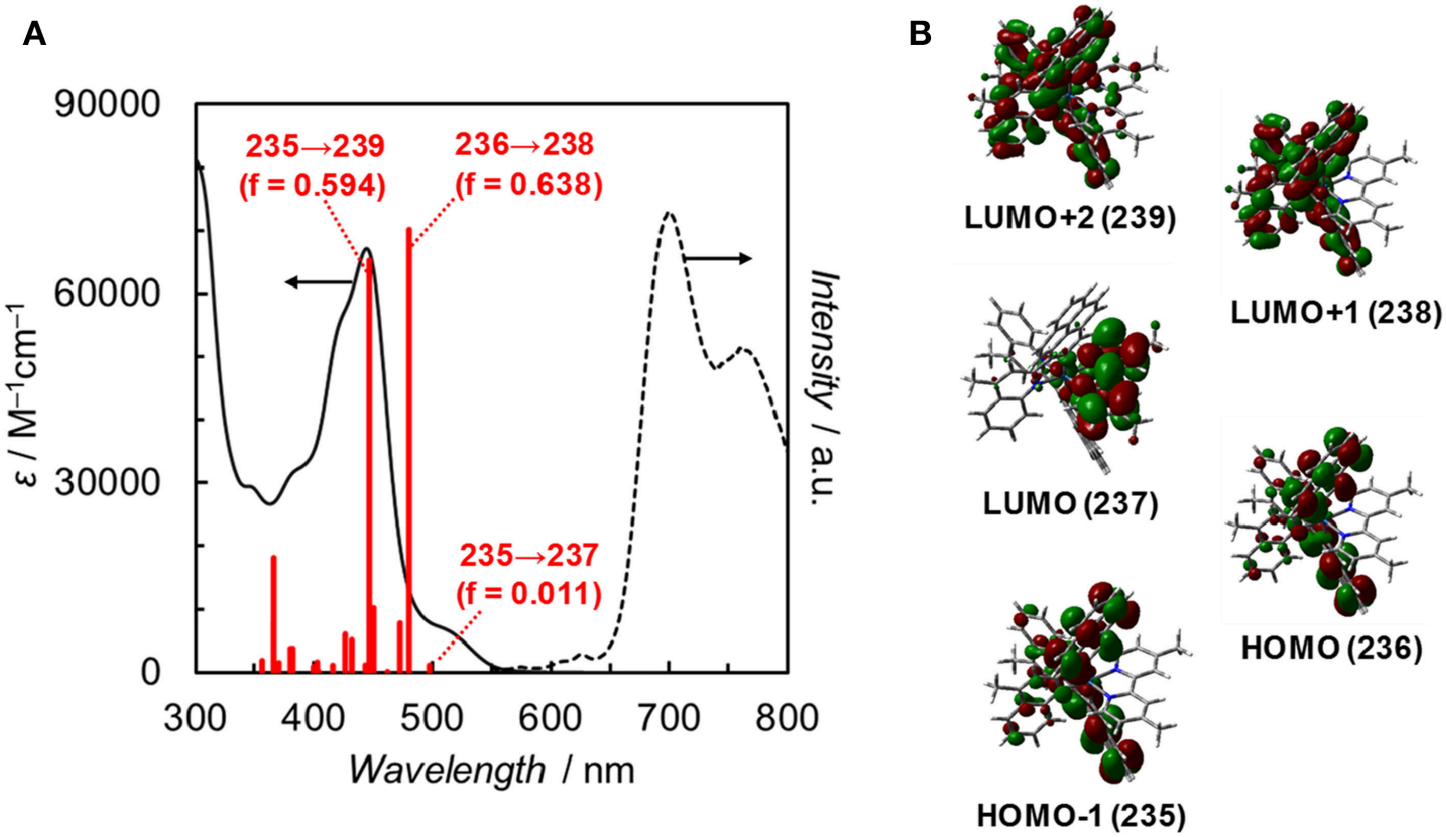
**(A)** UV–vis absorption spectrum (solid line), TDDFT theoretical excitations (red bars, >350 nm, f: oscillator strength), and emission spectrum (dotted line) of **Ir(pyr)** in DMA at 298 K. **(B)** Frontier orbitals of **Ir(pyr)**. Calculation method: B3LYP/LANL2DZ(Ir)/6-31G(d,p) (H, C, N) levels by using polarizable continuum model (PCM) with the default for DMA, isovalue = 0.02.

Fan et al. reported the photophysical and electrochemical properties of **Ir(pyr)** in dichloromethane or tetrahydrofuran (Fan et al., 2014). Since these solvents are less polar than the solvents suitable for CO_2_ reduction, such as *N,N*-dimethylacetamide (DMA), we measured the photophysical and electrochemical properties of **Ir(pyr)** in DMA (Kuramochi et al., 2014). Figure 3 shows the ultraviolet–visible (UV–vis) absorption spectrum of **Ir(pyr)** in DMA, which shows a much stronger absorption in the visible region (ε_444nm_ = 67,000 M^−1^ cm^−1^) compared to [Ir(piq)_2_(dmb)]^+^ and [Ru(dmb)_3_]^2+^. According to the time-dependent density functional theory (TD-DFT) calculation of the UV–vis spectrum of **Ir(pyr)** in DMA (Figure 4, red bars), the strong absorption band at 444 nm is due to the transitions from the highest occupied molecular orbital (HOMO)-1 to the lowest unoccupied molecular orbital (LUMO)+2 and from HOMO to LUMO+1, which correspond to the π−π^*^ transitions of the pyrene moieties. The absorption at a wavelength >500 nm is assigned to the transition from HOMO-1 to LUMO and corresponds to the transition from the interligand transition from the dmb to the pyrene moieties and might include some contribution from the singlet–triplet transitions, as described in the literature (Fan et al., 2014). The emission spectrum of **Ir(pyr)** in DMA is shown in Figure 4, and the Franck–Condon line-shape analysis (Figure S1) gave a 0–0 band energy gap of 14,500 cm^−1^ for **Ir(pyr)**, which is lower than that for [Ir(piq)_2_(dmb)]^+^ (16,950 cm^−1^) (Kuramochi and Ishitani, 2016).

The redox potentials of **Ir(pyr)** in DMA were obtained by cyclic voltammetry (CV; Figure S2) and differential pulse voltammetry (DPV) and are summarized in Table 1 together with those of [Ir(piq)_2_(dmb)]^+^ and **Ru** (Kuramochi and Ishitani, 2016; Kuramochi et al., 2018b). The oxidation waves of the Ir complexes were measured in acetonitrile due to its wide-potential window. The CV showed three each of reversible cathodic and irreversible anodic waves, indicating that **Ir(pyr)** is stable against reduction but relatively unstable to oxidation on the CV timescale. According to the DFT calculation, the LUMO mainly distributes across the dmb ligand of **Ir(pyr)** (Figure 4). Thus, it is expected that the first electron is injected into the dmb ligand, which benefits the electron transfer from the one-electron reduced species of **Ir(pyr)** to the **Ru** moiety. This property has been previously observed in [Ir(piq)_2_(dmb)]^+^ (Kuramochi and Ishitani, 2016).

**Table 1:** Electrochemical data in DMA (dichloromethane or THF) at 298 K^a^.

	**$E_{\text{1/2}}{}^{{}^{\circ}\text{x}}/V^{b}$**	**$E_{\text{1/2}}{}^{\text{red}}/V$**	*E*(PS^·^^+^/PS^*^)/V^c^	*E*(PS^*^/PS^·^^−^)/V^c^
[Ir(piq)_2_(dmb)]^+^^d^	+0.92	−1.75, −2.07, −2.31	−1.18	+0.35
**Ir(pyr)**	+0.84	−1.68, −1.86, −2.15	−0.96	+0.12
	(+0.45)^e^	(−1.70, −1.89, −2.29)^e^	–	(+0.23)^e^
**Ru**	–	−1.66^f^	–	–

a *E vs. Ag/AgNO_3_ (10 mM). ^b^Estimated by DPV in acetonitrile*.

c Excited-state oxidation and reduction potentials of the photosensitizer were calculated from E${}_{1/2}^{∙\text{x}}$ – E_00_ and E${}_{1/2}^{\text{red}}$ + E_00_, respectively.

d Kuramochi and Ishitani (2016).

e The values were correlated by using conversion factor (−0.631 V) from NHE to Ag/AgNO_3_, see Fan et al. (2014) and Elgrishi et al. (2017).

f *Kuramochi et al. (2018b)*.

### Emission Quenching by Electron Donors

The emission intensity of **Ir(pyr)–Ru** was similar to that of **Ir(pyr)**, suggesting that oxidative quenching of the excited state of the **Ir** unit by the **Ru** unit does not proceed in **Ir(pyr)–Ru**. This is reasonable because the oxidative quenching process is endothermic; the oxidation potential of the excited state of **Ir(pyr)** is much more positive (−0.96 V; Table 1) than the reduction potential of **Ru** (−1.66 V).

**Figure 5:**
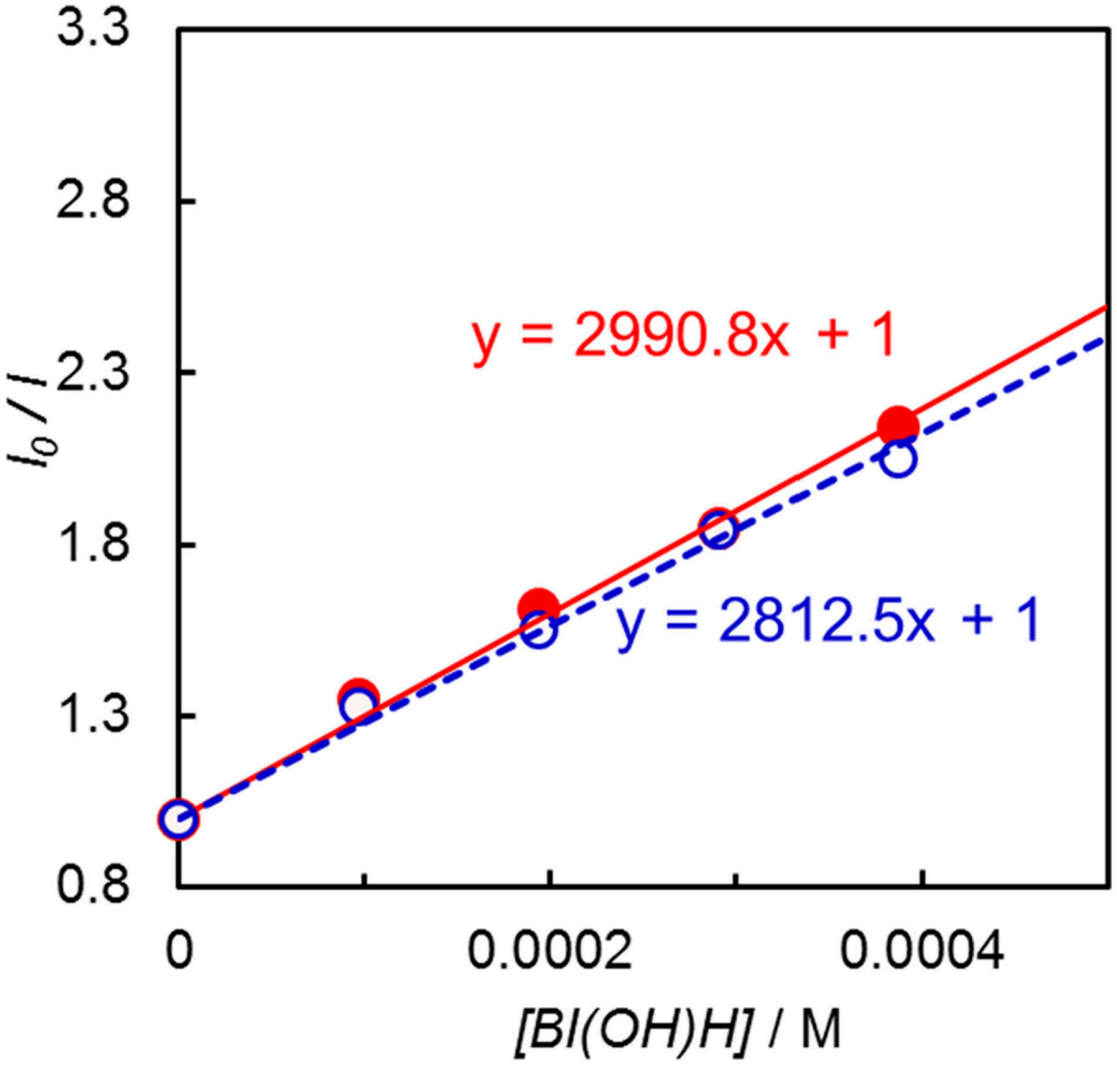
Emission quenching of the excited **Ir(pyr)** (closed red circle) and **Ir(pyr)–Ru** (open blue circle) by BI(OH)H in DMA/TEOA (5:1 v/v) at 298 K.

Emission quenching of **Ir(pyr)** by 1-benzyl-1,4-dihydronicotinamide (BNAH) was inefficient: Stern–Volmer constant (*K*_SV_) = 13 M^−1^ in DMA. Assuming that the emission lifetime is 3.1 μs (Fan et al., 2014), the quenching rate constant (*k*_q_) was estimated to be 4.2 × 10^6^ M^−1^ s^−1^. When a stronger electron donor, BI(OH)H (Hasegawa et al., 2006; Tamaki et al., 2015), was used, the emission of **Ir(pyr)** was more efficiently quenched (Figure 5); *K*_SV_ reached 3,000 M^−1^ in DMA/TEOA (5:1 v/v), and *k*_q_ was 9.7 × 10^8^ M^−1^ s^−1^, which is close to the diffusion-controlled rate constant (Tamaki et al., 2013). The *K*_SV_ of **Ir(pyr)–Ru** by BI(OH)H was 2,800 M^−1^, which is similar to that of **Ir(pyr)**. In previous work by Fan et al. (2014), **Ir(pyr)** did not work as a PS for H_2_ evolution because the emission from **Ir(pyr)** was not quenched by TEA. This emission is not quenched by TEOA as well because TEOA has a similar oxidation potential to TEA. Conversely, BI(OH)H significantly quenches the emission from **Ir(pyr)**, indicating efficient electron transfer from BI(OH)H to the excited **Ir(pyr)**.

### Photocatalytic CO_2_ Reduction

**Figure 6:**
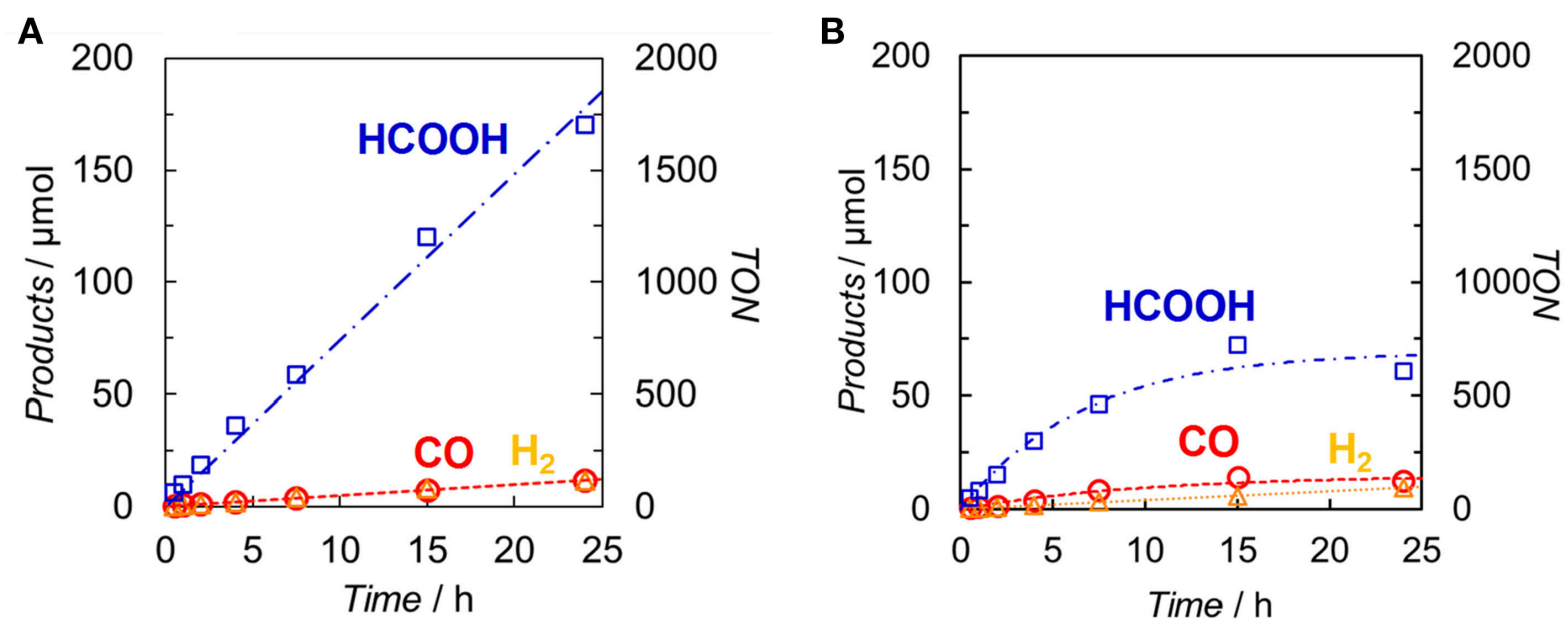
Time dependences of the products during the photo-irradiation of CO_2_-saturated DMA/TEOA (5:1 v/v, 2.0 ml) solutions containing **(A)** a mixed system of **Ir(pyr)** (0.05 mM) and **Ru** (0.05 mM) or **(B) Ir(pyr)–Ru** (0.05 mM) in the presence of BI(OH)H (0.1 M): CO (°), HCOOH (□) and H_2_ (Δ). A 500-W high-pressure Hg lamp was used for the irradiation (λ > 480 nm).

DMA-TEOA (5:1 v/v) mixed solutions containing both **Ir(pyr)** and **Ru** or **Ir(pyr)–Ru** (0.05 mM) as the photocatalysts and BI(OH)H as the ED were irradiated at λ_ex_ > 480 nm under a CO_2_ atmosphere. In both cases, HCOOH was mainly detected with small amounts of CO and H_2_. Figure 6 shows the time profiles of product formation during the photocatalytic reaction. Blank experiments in the absence of the **Ru** catalyst produced trace amounts of CO (3.9 μmol) and formate (7.6 μmol) after 24 h. From the Stern–Volmer constants, the quenching efficiencies of the excited states of **Ir(pyr)** and **Ir(pyr)–Ru** by 0.1 M BI(OH)H were estimated as η_q_ > 99%, indicating that the excited states of **Ir(pyr)** and **Ir(pyr)–Ru** were almost completely quenched by BI(OH)H under these reaction conditions. Figure 6 shows that **Ir(pyr)** does work as a PS for CO_2_ reduction when using BI(OH)H. The time profiles for the mixture of **Ir(pyr)** and **Ru** showed a linear increase reaching a TON_HCOOH_ = ~2,000 during 24-h irradiation, indicating that **Ir(pyr)** has a high durability during photocatalytic CO_2_ reduction. Although **Ir(pyr)–Ru** also worked as a photocatalyst for CO_2_ reduction, it showed a lower activity compared to the mixture of **Ir(pyr)** and **Ru**. While the initial formation rates of the products were similar, the reaction stopped after just 5 h of irradiation in the case of **Ir(pyr)–Ru**. Because the reaction solution of **Ir(pyr)–Ru** was decolorized during the photocatalytic reaction, the low activity of **Ir(pyr)–Ru** would result from its low durability. The decoloration was also observed in irradiation experiments of [Ir(piq)_2_(dmb)]^+^ and ED without the CAT because of hydrogenation of the ligands in [Ir(piq)_2_(dmb)]^+^ (Kuramochi and Ishitani, 2016). Thus, it is also expected that the decoloration of **Ir(pyr)–Ru** is caused by hydrogenation of the Ir unit. In **Ir(pyr)–Ru**, the accumulated electron(s) might be stabilized because of electron hopping between the Ir and Ru units, which might be enhanced by the hydrogenation of the Ir unit.

**Figure 7:**
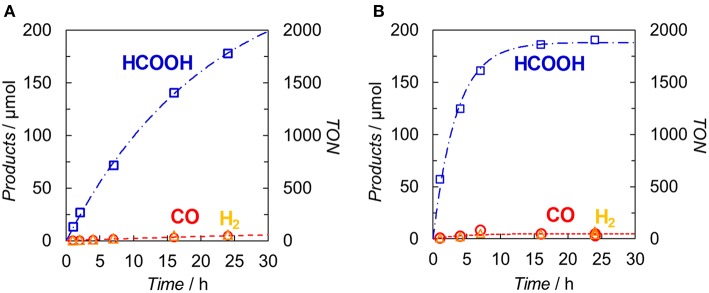
Time dependences of the products during the photo-irradiation of CO_2_-saturated DMA/TEOA (5:1 v/v, 2.0 ml) solutions containing **(A) Ir(pyr)** (0.05 mM) or **(B)** [Ru(dmb)_3_]^2+^ (0.05 mM) in the presence of *trans*-Ru(bpy)(CO)_2_Cl_2_ (0.05 mM) and BI(OH)H (0.1 M): CO (°), HCOOH (□) and H_2_ (Δ). A 500-W high-pressure Hg lamp was used for the irradiation (λ > 480 nm).

Figure 7 illustrates the time profiles of the products during the irradiation of CO_2_-saturated DMA/TEOA (5:1 v/v, 2.0 ml) solutions containing **Ir(pyr)** or [Ru(dmb)_3_]^2+^ as PS in the presence of *trans*-Ru(bpy)(CO)_2_Cl_2_ (bpy = 2,2-bipyridine) and BI(OH)H as CAT and ED, respectively. The initial formation rate of HCOOH in the system using **Ir(pyr)** (Figure 7A) is slower than that using [Ru(dmb)_3_]^2+^ (Figure 7B), although **Ir(pyr)** has more intense absorption band at >480 nm than [Ru(dmb)_3_]^2+^ (Figure 3). In Figure 7, *trans*-Ru(bpy)(CO)_2_Cl_2_ is used instead of **Ru**. Although *trans*-Ru(bpy)(CO)_2_Cl_2_ has a much less negative reduction potential (−1.51 V vs. Ag/AgNO_3_; Kuramochi et al., 2015) than **Ru** (−1.66 V *vs*. Ag/AgNO_3_), the initial formation rate of HCOOH in the system using *trans*-Ru(bpy)(CO)_2_Cl_2_ (Figure 7A) is similar to that using **Ru** (Figure 6A), suggesting that the electron transfer process from the one-electron reduced **Ir(pyr)** to CAT is not the rate-determining step and does not significantly affect the reaction rate. Considering that the emission of **Ir(pyr)** is almost completely quenched, the slow initial formation rate of HCOOH in **Ir(pyr)** would result from the competitive back-electron transfer process soon after the electron transfer from BI(OH)H to the excited state of **Ir(pyr)** in the solvent cage (Kavarnos, 1993; Nakada et al., 2015).

## Conclusion

Photocatalytic CO_2_ reduction using **Ir(pyr)** as PS, which has a strong absorption in the visible region, proceeded efficiently for more than 1 day when a suitable electron donor, BI(OH)H, and **Ru** were used as CAT. A new supramolecular photocatalyst, **Ir(pyr)–Ru**, was successfully synthesized, which exhibited a similar reaction rate during the initial stage of CO_2_ reduction to that of the mixed system, but the durability of **Ir(pyr)–Ru** was lower than that of the mixed system. While Ir(pyr) showed high durability in the mixed system, the initial formation rate of HCOOH tended to be slower than that of the catalytic system using [Ru(dmb)_3_]^2+^ as PS, which is possibly due to the faster back-electron transfer from the reduced Ir(pyr) to the oxidized BI(OH)H.

## Experimental Section

### General Procedure

All chemicals and solvents were of commercial reagent quality and were used without further purification unless otherwise stated. DMA was dried over molecular sieves of size 4 Å and distilled under reduced pressure. TEOA was distilled under reduced pressure. Tetraethylammonium tetrafluoroborate was dried *in vacuo* at 100°C overnight before use. [Ir(piq)_2_(BL)](PF_6_) (Kuramochi and Ishitani, 2016), BNAH (Mauzerall and Westheimer, 1955), BI(OH)H (Hasegawa et al., 2005; Zhu et al., 2008), **Ru** (Anderson et al., 1995), and BL (Sun et al., 1997) were synthesized according to literature procedures. ^1^H NMR spectra were recorded on an AL400 NMR spectrometer. IR spectra were measured in dichloromethane on a JASCO FT/IR-610 spectrometer. Electrospray ionization–mass spectroscopy (ESI-MS) was undertaken using a SHIMADZU LCMS-2010A system with acetonitrile as a mobile phase. UV–vis absorption spectra were recorded with a JASCO V-670 instrument. Emission spectra were measured at 25°C under an Ar atmosphere using a JASCO FP-8600 spectrofluorometer with correlation for the detector sensitivity. Emission quenching experiments were performed in DMA or DMA/TEOA (5:1 v/v) solutions containing a complex and several different concentrations of BNAH or BI(OH)H.

### Emission Spectral Fitting

Double-mode Franck–Condon band shape analysis was used to fit the emission spectra. The spectral fittings were carried out according to the following equation (Caspar et al., 1984) using the Wavemetrics Igor software.

(1) $$\begin{array}{l} I\left(\tilde{v}\right)=\sum_{n_{1}=0}^{5}\sum_{n_{2}=0}^{5}{\left(\frac{E_{00}-n_{1}{\tilde{v}}_{1}-n_{2}{\tilde{v}}_{2}}{E_{00}}\right)}^{4}\left(\frac{S_{1}^{n_{1}}}{n_{1}!}\right)\left(\frac{S_{2}^{n_{2}}}{n_{2}!}\right)\\ \;\exp\left[-4log2{\left(\frac{\tilde{v}-E_{00}+n_{1}{\tilde{v}}_{1}+n_{2}{\tilde{v}}_{2}}{{\tilde{v}}_{1/2}}\right)}^{2}\right] \end{array}$$

*I*(ν) is the relative emission intensity at frequency ν. *E*_00_ is the energy gap between the zeroth vibrational levels in the ground and excited states, *n*_1_ and *n*_2_ are the vibrational quantum numbers of the high- and low-frequency vibrational modes, respectively, *S*_1_ and *S*_2_ are the Huang–Rhys factors, and ν_1/2_ is the half-width at half-maximum (fwhm) of the individual vibronic band. The 0–0 band energy gaps between the lowest excited state and the ground state were obtained from the emission spectral fitting (Figure S1).

### [Ir(pyr-mq)_2_(CH_3_CN)_2_](PF_6_)

[(pyr-mq)_2_Ir-μ-Cl]_2_ (100 mg, 5.4 × 10^−5^ mol), AgPF_4_ (31 mg, 1.2 × 10^−4^ mol), and acetonitrile (10 ml) were placed in a 50-ml flask. The mixture was stirred for 4 h at room temperature. The resulting suspension was filtered through Celite pad to remove AgCl. The filtrate was concentrated to *ca*. 1 ml, and the product was precipitated by the addition of diethyl ether (10 ml). After cooling the suspension for 30 min at 0°C, the product was collected by filtration, giving 96 mg (80%) of the titled compound as a dark orange solid: ^1^H NMR (CDCl_3_, 400 MHz) δ 9.18 (brs, 2H), 8.73 (d, *J* = 8.8 Hz, 2H), 8.56 (s, 2H), 8.23 (d, *J* = 7.2 Hz, 2H), 8.15–7.95 (m, 8H), 7.85–7.70 (m, 6H), 7.27 (m, 2H), 6.40 (brs, 2H), 3.23 (s, 6H), 2.22 (s, 6H, C*H*_3_CN).

### [Ir(pyr-mq)_2_(BL)](PF_6_)

The bridging ligand (BL, 80 mg, 2.2 × 10^−4^ mol), dichloromethane (40 ml), and methanol (20 ml) were placed in a 100-ml flask, and the system was purged with argon gas. A solution of [Ir(pyr-mq)_2_(CH_3_CN)_2_](PF_6_) (80 mg, 7.3 × 10^−5^ mol) in dichloromethane (10 ml) and methanol (5 ml) was then added dropwise at room temperature. The reaction mixture was stirred at room temperature. The reaction progress was monitored with ESI-MS. After stirring for 48 h, the resulting solution was evaporated. The residue was purified with a silica gel column (1 to 2 vol% methanol in dichloromethane). The second red band eluted with 2 vol% methanol was collected and evaporated to dryness, giving 42 mg (43% based on the iridium precursor) of the titled complex as a dark red solid: ESI-MS *m/z*: 1,243 ([M–PF${}_{6}^{-}$]^+^). ^1^H NMR (CDCl_3_, 400 MHz) δ 8.94 (brs, 1H), 8.92 (brs, 1H), 8.60 (d, *J* = 2.8 Hz, 2H), 8.51 (d, *J* = 5.2 Hz, 1H), 8.45 (d, *J* = 5.2 Hz, 1H), 8.24–8.12 (m, 8H), 8.02 (s, 1H), 8.00 (s, 1H), 7.91–7.76 (m, 8H), 7.58 (s, 2H), 7.52–7.38 (m, 5H), 7.35 (m, 1H), 7.25–7.21 (m, 1H), 7.09 (d, *J* = 4.4 Hz, 1H), 6.99–6.88 (m, 3H), 6.77 (m, 1H), 3.13–3.00 (m, 4H), 2.97 (s, 3H), 2.95 (s, 3H), 2.45 (s, 3H), 2.42 (s, 3H).

### Ir(pyr)–Ru

An acetone/ethenol (1:2 v/v) mixed solution (6 ml) containing [Ru(CO)_2_Cl_2_]_n_ (9.9 mg, 4.3 × 10^−5^/n mol) was refluxed for 1 h, and then [Ir(pyr-mq)_2_(BL)](PF_6_) (30 mg, 2.2 × 10^−5^ mol) was added to it. The reaction mixture was heated to 60°C and stirred for 4.5 h under Ar atmosphere. As the reaction proceeded, the starting red solution became a red suspension. The resulting solid was filtered and washed with ethanol. The solid was dissolved in dichloromethane (*ca*. 2 ml) and filtered to remove insoluble materials. The solution was evaporated to afford 30 mg (87%) of the titiled compound as a dark red solid. ESI-MS *m/z*: 1,471 ([M–PF${}_{6}^{-}$]^+^). FT-IR ν_CO_/cm^−1^: 1,992, 2,058. Anal. calcd (%) for C_78_H_54_Cl_2_F_6_IrN_6_O_2_PRu· 3H_2_O: C, 56.08; N, 5.03; H, 3.62. Found (%): C, 55.88; N, 4.81; H, 3.21. ^1^H NMR (CDCl_3_, 400 MHz) δ 8.97–8.88 (m, 4H), 8.74 (brs, 1H), 8.65–8.62 (m, 3H), 8.42 (brs, 1H), 8.30 (brs, 1H), 8.25–8.21 (m, 2H), 8.18–8.15 (m, 2H), 8.03–8.01 (m, 2H), 7.96–7.79 (m, 8H), 7.61 (s, 1H), 7.56 (s, 1H), 7.53–7.49 (m, 2H), 7.45–7.40 (m, 4H), 7.36 (m, 1H), 7.28–7.24 (m, 1H), 7.09 (d, *J* = 5.6 Hz, 1H), 6.98–6.88 (m, 3H), 3.05–2.94 (m, 4H), 2.99 (s, 3H), 2.98 (s, 3H), 2.61 (s, 3H), 2.47 (s, 3H).

### Photocatalytic CO_2_ Reduction

DMA-TEOA (2 ml; 5:1 v/v) solutions containing a mixture of PS and CAT or the supramolecular **Ir(pyr)–Ru** complex and BI(OH)H were bubbled with CO_2_ for 30 min. Photo-irradiations were carried out in 11-mL Pyrex tubes (i.d. = 8 mm) with light at λ > 480 nm using a 500-W high-pressure Hg lamp combined with a K_2_CrO_4_ solution filter (30% w/w, optical path length: 1 cm) using a merry-go-round apparatus. The reaction temperature was maintained at 25°C using an IWAKI constant-temperature system (CTS-134A). The gaseous reaction products (CO and H_2_) were quantified with GC-TCD (GL Science GC323), and the product (formate) in the solutions was analyzed with a capillary electrophoresis system (Otuka Electronics Co.CAPI-3300I).

### Computational Methods

DFT calculations were carried out using the Gaussian 09 package of programs (Frisch et al., 2009). Each structure was fully optimized using the B3LYP functional using the 6-31G(d,p) basis set for all atoms except Ir and the standard double-ζ type LANL2DZ basis set with the effective core potential of Hay–Wadt for Ir. The calculation was carried out by using the polarizable continuum model (PCM) with default parameter for DMA. The stationary points were verified using the vibrational analysis.

## Author Contributions

YK concieved the research and conducted experiments. OI directed the project and co-wrote the paper.

### Conflict of Interest Statement

The authors declare that the research was conducted in the absence of any commercial or financial relationships that could be construed as a potential conflict of interest.
