# Supplementary material for: An Ir(III) Complex Photosensitizer With Strong Visible Light Absorption for Photocatalytic CO2 Reduction
Source: Front Chem. 2019 May 1;7:259. doi: 10.3389/fchem.2019.00259 (PMC6504785; doi:10.3389/fchem.2019.00259)
Supplement: Supplementary file 1 [file Data_Sheet_1.pdf]

Supplementary Information

An Ir(III) Complex Photosensitizer with Strong Visible Light Absorption for Photocatalytic CO_2 Reduction

Yusuke Kuramochi[a, b]* and Osamu Ishitani[a]*

[a] Department of Chemistry, Graduate School of Science and Engineering, Tokyo Institute of Technology, 2-12-1-NE-1, O-okayama, Meguro-ku, Tokyo, 152-8550, Japan
[b] Present address: Department of Chemistry, Faculty of Science Division II, Tokyo University of Science, 1-3, Kagurazaka, Shinjuku-ku, Tokyo, 162-8601, Japan

Synthetic Procedures

2-(Pyren-1-yl)-4-methylquinoline (pyr-mq).[S1] Pyren-1-yl-1-boronic acid (1.1 g, 4.3×10^{-3} mol), 2-chloro-4-methylquinoline (510 mg, 2.9×10^{-3} mol) and Na_2CO_3 (2.1 g, 2.0×10^{-2} mol) were dissolved in a mixed solvent of toluene (30 mL), ethanol (10 mL) and water (10 mL). The solution was degassed by freeze-thaw cycles. The flask was purged with Ar gas and $Pd(PPh_3)_4$ (330 mg, 2.9×10^{-4} mol) was added. The reaction mixture was heated to reflux and stirred for 48 h. Water (50 mL) was added to the resulting solution and the product was extracted with diethyl ether (50 mL×3). The organic layer was collected and dried over anhydrous Na_2SO_4. The residue obtained by evaporation of the solvent was purified with a silica gel column (EtOAc/hexane 1:20 → 1:4). The pale-yellow second fraction was collected and evaporated to afford 780 mg (80%, lit. 99%[S1]) of the titled compound as a pale yellow solid: TLC (silica gel, EtOAc/hexane 1:9) $R_f = 0.3$; ^{1}H NMR (400 MHz, $CDCl_3$) δ 8.43 (d, $J = 9.2$ Hz, 1H), 8.29-8.24 (m, 3H), 8.21-8.16 (m, 2H), 8.11-7.99 (m, 5H), 7.79 (m, 1H), 7.68 (s, 1H), 7.63 (m, 1H), 2.81 (s, 3H).

[(pyr-mq)$_2$Ir-*μ*-Cl]$_2$.[S1] In a 100 mL flask were placed $IrCl_3\cdot3H_2O$ (140 mg, 3.9×10^{-4} mol), 2-(pyren-1-yl)-4-methylquinoline (300 mg, 8.7×10^{-4} mol), 2-ethoxyethanol (18 mL) and water (6 mL). The mixture was heated to reflux and stirred for 45 h. The dark-red precipitate was collected by filtration and washed with methanol (10 mL×3). The solid was dried to give 210 mg (59%, lit. 47%[S1]) of the titled compound as a dark-red solid.

[Ir(pyr-mq)$_2$(dmb)](PF$_6$).[S1] In a 50 mL flask were placed [(pyr-mq)$_2$Ir-*μ*-Cl]$_2$ (95 mg, 5.2×10^{-5} mol), 4,4'-dimethyl-2,2'-bipyridine (19 mg, 1.0×10^{-4} mol) and ethylene glycol (5 mL). The reaction mixture was refluxed for 22 h and allowed to cool to room temperature. After water (*ca.* 40 mL) was added to it, a aqueous solution (15 mL) containing KPF_6 (2.0 g) was added to precipitate the product. The solid was collected by filtration and washed with water (100 mL). The dark-red solid was recrystalized from CH_2Cl_2 and diethyl ether to obtain 67 mg (54%, lit. 55%[S1]) of the titled compound: ^{1}H NMR ($CDCl_3$, 400 MHz) δ 8.96 (d, $J = 9.6$ Hz, 2H), 8.64 (s, 2H), 8.24 (d, $J = 9.6$ Hz, 2H), 8.17 (d, $J = 6.8$ Hz, 2H), 8.09 (s, 2H), 8.03 (d, $J = 6.8$ Hz, 2H), 7.94-7.89 (m, 4H), 7.86 (d, $J = 6.0$ Hz, 2H), 7.81 (d, $J = 8.8$ Hz, 2H), 7.62 (s, 2H), 7.54 (d, $J = 9.6$ Hz, 2H), 7.47-7.42 (m, 4H), 7.01-6.94 (m, 4H), 2.98 (s, 6H), 2.43 (s, 6H).

Reference

[S1] S. Fan, X. Zong, P. E. Shaw, X. Wang, Y. Geng, A. R. G. Smith, P. L. Burn, L. Wang, S.-C. Lo, *Phys.Chem.Chem.Phys.*, 2014, **16**, 21577−21585.

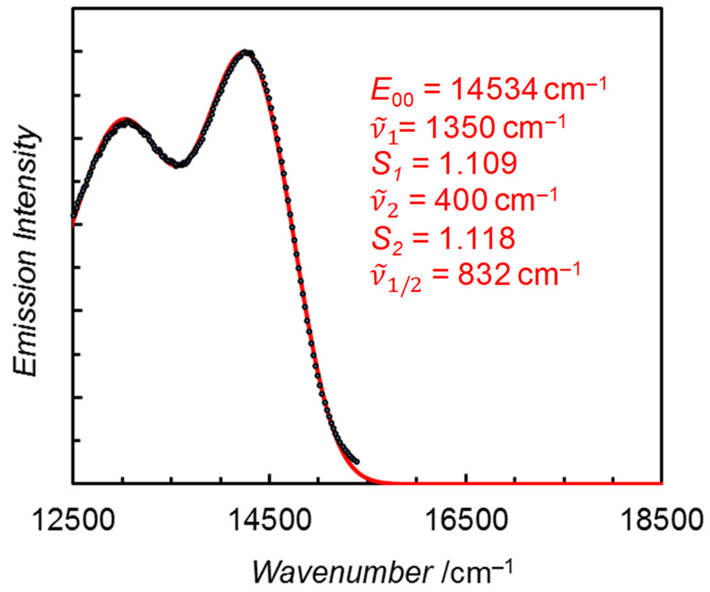

Figure S1. Corrected emission spectrum of **Ir(pyr)** in DMA at 298 K and simulation curve (red line) by mean of double-mode Franck-Condon line-shape analysis.

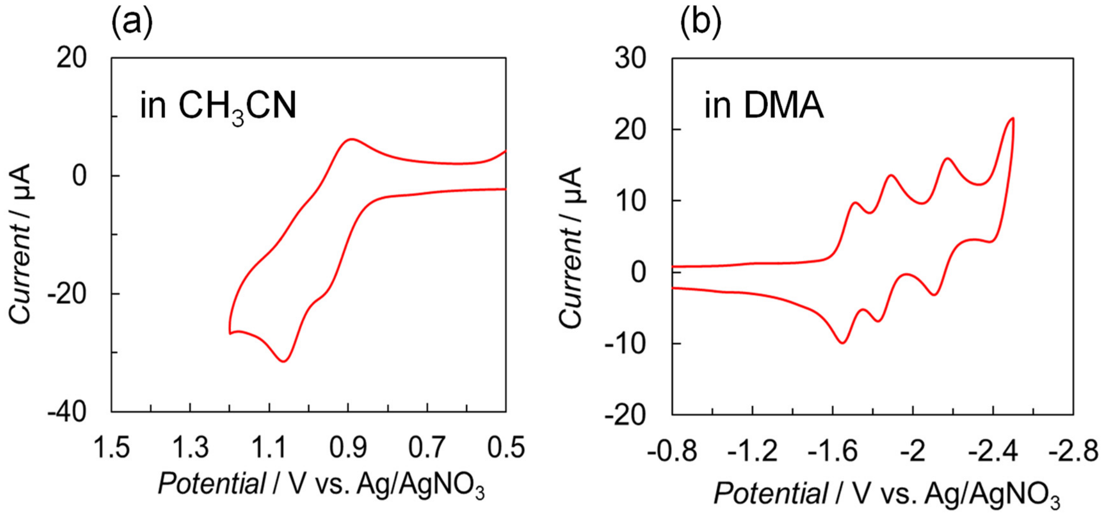

Figure S2. CVs of **Ir(pyr)** (0.5 mM) under Ar-saturated (a) acetonitrile and (b) DMA with 0.1 M Et_4NBF_4 as the supporting electrolyte and Ag/$AgNO_3$ (10 mM, in acetonitrile or DMA) as the reference electrode. Scan rate: 200 mVs^{-1}.
